# Association of RSV Vaccination With Clinical Outcomes in Older Adults With Heart Failure: A Propensity‐Matched Analysis

**DOI:** 10.1002/clc.70488

**Published:** 2026-10-06

**Authors:** Allahdad Khan, Muhammad Raffey Shabbir, Jamil Nasrallah, Mahrukh Imtiaz, Harshith Thyagaturu, Marat Fudim, Robert J. Mentz, Gregg C. Fonarow

**Affiliations:** ^1^ Department of Medicine Nishtar Medical University Multan Pakistan; ^2^ Department of Internal Medicine Marshfield Clinic, Sanford Health Marshfield Wisconsin USA; ^3^ Department of Medicine, Faculty of Medical Sciences Lebanese University Beirut Lebanon; ^4^ Department of Cardiology West Virginia University Morgantown West Virginia USA; ^5^ Department of Medicine, Division of Cardiology Duke University School of Medicine Durham North Carolina USA; ^6^ Department of Medicine, Division of Cardiology Geffen School of Medicine at UCLA Los Angeles California USA

## Abstract

**Background:**

Respiratory syncytial virus (RSV) is increasingly recognized as a trigger for adverse outcomes in older adults with heart failure (HF). Following FDA approval of RSV vaccines for adults ≥ 60 years in 2023, real‐world evidence in HF populations remains limited.

**Methods:**

We conducted a multicenter retrospective cohort study using the TriNetX US Collaborative Network. Adults ≥ 60 years with HF were identified and stratified by RSV vaccination status from May 1, 2023, to January 01, 2026. Propensity score matching (1:1) was performed. The primary outcome was all‐cause mortality. Secondary outcomes included respiratory failure, healthcare utilization, and cardiovascular events.

**Results:**

After PSM, 777 vaccinated and 777 unvaccinated HF patients were analyzed. All‐cause mortality (HR 0.51, 95% CI, 0.33−0.79) and ICU admissions were significantly lower in vaccinated patients (2.7% vs. 5.8%, HR 0.46, 95% CI 0.25–0.86), while healthcare utilization was significantly higher in the vaccinated cohort (HR 1.85, 95% CI 1.65–2.08). No significant differences were observed in respiratory failure (7.3% vs. 9.4%, HR 0.87, 95% CI 0.61–1.23), inpatient or emergency visits (29.9% vs. 35.1%, HR 0.97, 95% CI 0.81–1.16), HF exacerbations, myocardial infarction, or stroke. Age‐stratified subgroup analyses (60–75 and 75–90 years) showed consistent findings, with no significant cardiovascular differences and significantly higher healthcare utilization in vaccinated patients across both age groups.

**Conclusions:**

In older adults with HF, RSV vaccination was associated with lower mortality and fewer ICU admissions, without a corresponding reduction in overall inpatient/emergency use or acute‐care utilization. These findings are hypothesis‐generating and may reflect prevention of severe respiratory illness.

## Graphical abstract

In older adults with heart failure, RSV vaccination was associated with lower all‐cause mortality (HR 0.51), fewer ICU admissions (HR 0.46), and increased healthcare utilization (HR 1.85), with no significant differences in cardiovascular outcomes or respiratory failure after propensity score matching.

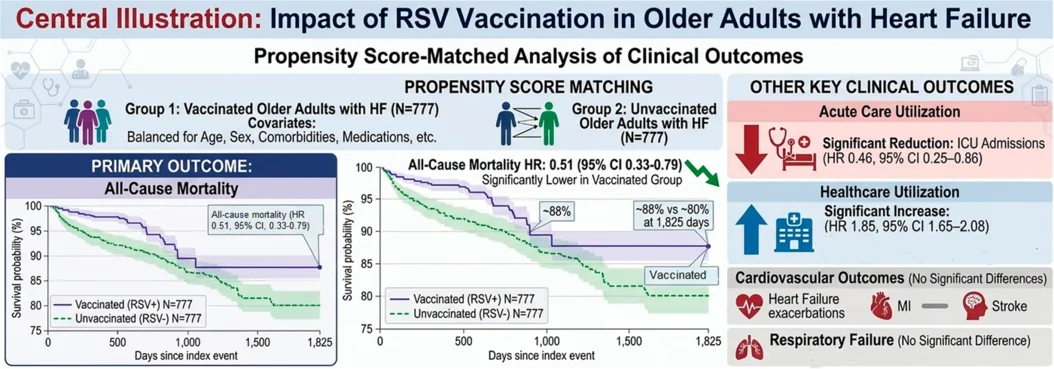

## 1 Introduction

Respiratory syncytial virus (RSV) is increasingly recognized as a major cause of lower respiratory tract infection (LRTI) among adults aged 60 years and older, resulting in substantial morbidity, hospitalization, and mortality [1]. Although historically considered a pediatric pathogen, RSV is now recognized as an important contributor to respiratory disease and healthcare utilization in older adults [2]. Individuals with advanced age and chronic comorbidities, particularly cardiovascular disease, are at greatest risk for severe infection and adverse clinical outcomes, highlighting the need for effective preventive strategies [3].

Patients with heart failure (HF) are especially vulnerable to complications following RSV infection. Respiratory viral infections may precipitate HF decompensation through systemic inflammation, increased metabolic demand, hypoxemia, and myocardial stress, leading to worsening cardiac function. Consistent with these mechanisms, RSV infection has been associated with increased risks of HF hospitalization, acute cardiovascular events, and mortality, making HF an important target population for RSV prevention [4, 5].

Recently approved RSV vaccines have demonstrated high efficacy against RSV‐associated lower respiratory tract disease in older adults in pivotal randomized controlled trials. However, patients with HF were underrepresented in these studies, and data on cardiovascular outcomes and real‐world vaccine effectiveness in this high‐risk population remain limited [6, 7, 8].

Real‐world studies complement randomized trials by evaluating vaccine effectiveness in broader patient populations encountered in routine clinical practice. Propensity score matching can reduce confounding by balancing baseline characteristics between vaccinated and unvaccinated individuals, providing more robust estimates of treatment effectiveness. Evaluating RSV vaccination in patients with HF may therefore provide important evidence to guide vaccination strategies in this vulnerable population.

Therefore, we conducted a propensity score‐matched real‐world cohort study to evaluate the association between RSV vaccination and clinical outcomes among older adults with HF. We hypothesized that RSV vaccination would be associated with reduced risks of all‐cause mortality, hospitalization, HF exacerbation, and other adverse clinical outcomes compared with no vaccination.

## 2 Methods

We conducted a multicenter retrospective cohort analysis using the TriNetX US Collaborative Network to evaluate the association between RSV vaccination and clinical outcomes among older adults with HF [9]. Eligible patients were required to be 60 years of age or older at their most recent recorded encounter and to have a diagnosis of HF (ICD‐10‐CM I50.x) documented within the preceding 5 years. Patients in the vaccinated cohort were additionally required to have documentation of RSV vaccination, identified using Current Procedural Terminology (CPT) codes 90678 or 90679 or the legacy ICD‐9‐CM vaccination code V04.82, administered on or after May 1, 2023 (corresponding to the timing of US FDA approval of RSV vaccines) through January 01, 2026. Patients were excluded if they had a history of congenital heart disease (ICD‐10‐CM Q24), diseases of the blood and blood‐forming organs or disorders involving the immune mechanism (ICD‐10‐CM D50–D89), or other rheumatic heart diseases (ICD‐10‐CM I09) documented at any time before cohort assignment (Table 1).

**Table 1:** Inclusion and exclusion criteria used in this analysis.

Inclusion criteria	Exclusion criteria
Age (at least 60 years [most recent occurrence])	UMLS:ICD10CM:Q24 Other congenital malformations of heart or UMLS:ICD10CM:D50‐D89 Diseases of the blood and blood‐forming organs and certain disorders involving the immune mechanism or UMLS:ICD10CM:I09 Other rheumatic heart diseases date constraint(The terms in this group occurred at any time)
BASELINE GROUP must have diagnosis UMLS:ICD10CM:I50 Heart failure (This group occurred 5 years ago)	
RSV Status (for the vaccinated group) must have any of UMLS:ICD9CM:V04.82 Need for prophylactic vaccination and inoculation against respiratory syncytial virus (RSV) or UMLS:CPT:90678 Respiratory syncytial virus vaccine, preF, subunit, bivalent, for intramuscular use or UMLS:CPT:90679 Respiratory syncytial virus vaccine, preF, recombinant, subunit, adjuvanted, for intramuscular use (The terms in this group occurred on or after May 1, 2023)	

Propensity score matching (1:1) was performed to balance demographic characteristics, comorbidities, and medication use between vaccinated and unvaccinated patients. Following matching, Cohort 1 (RSV‐vaccinated) and Cohort 2 (unvaccinated) each included 777 patients with well‐balanced baseline characteristics. After completing the matching process, we calculated a standardized difference to evaluate the balance of patient characteristics between the matched cohorts. A standardized difference < 0.1 indicates that the groups are well‐balanced.

All analyses were performed using the TriNetX platform integrated analytics tools, accessed on December 28, 2025, with additional analyses conducted using the R survival package (version 3.2‐3). Baseline characteristics between the RSV‐vaccinated and unvaccinated cohorts were compared using the chi‐square test for categorical variables and the two‐sample *t*‐test for continuous variables. Propensity score matching (1:1) was performed using demographic characteristics, clinical comorbidities, and medication variables to minimize baseline differences between cohorts. Subgroup analysis was done for age ranges 60−75 and 75−90 to check for age‐related differences.

For clinical outcomes, absolute risks, risk differences (RDs), risk ratios (RRs), and odds ratios (ORs) were calculated. Time‐to‐event outcomes were evaluated using Kaplan–Meier survival analysis with log‐rank testing, and Cox proportional hazards models were used to estimate hazard ratios (HRs) with 95% confidence intervals (CIs). Statistical significance was defined as a two‐sided *p* < 0.05. Because this study used de‐identified electronic health record data from the TriNetX network, it qualified for exemption from institutional review board review.

## 3 Results

A total of 4139 HF patients met initial criteria (Figure 1). After PSM (Table 2, Figure 2), we included 777 vaccinated and 777 unvaccinated patients. The RSV‐vaccinated and unvaccinated cohorts were highly comparable across all measured baseline characteristics. After PSM, the mean age and standard deviation were 70.6 ± 8.73 with a median of 68 and an interquartile range (IQR) of 15 for the RSV‐vaccinated cohort and 70.6 ± 8.78 with a median of 70 and an IQR of 14 for the RSV‐unvaccinated cohort. More details regarding the age of included patients are in Figure 3. In terms of ethnicity, 69.2% of patients identified as Not Hispanic or Latino, while 6.6% of patients identified as Hispanic or Latino, and 24.2% of patients refused to identify their ethnicity. In terms of Race, mainly 77.3% of patients were White, followed by 14.2% of patients who were Black or African American, 3.6% of patients were Asian, 1.28% were American Indian or Alaska Native, and 1.28% of patients were Native Hawaiian or other Pacific Islanders. The remaining patients were identified as another race or had an unknown race.

**Figure 1:**
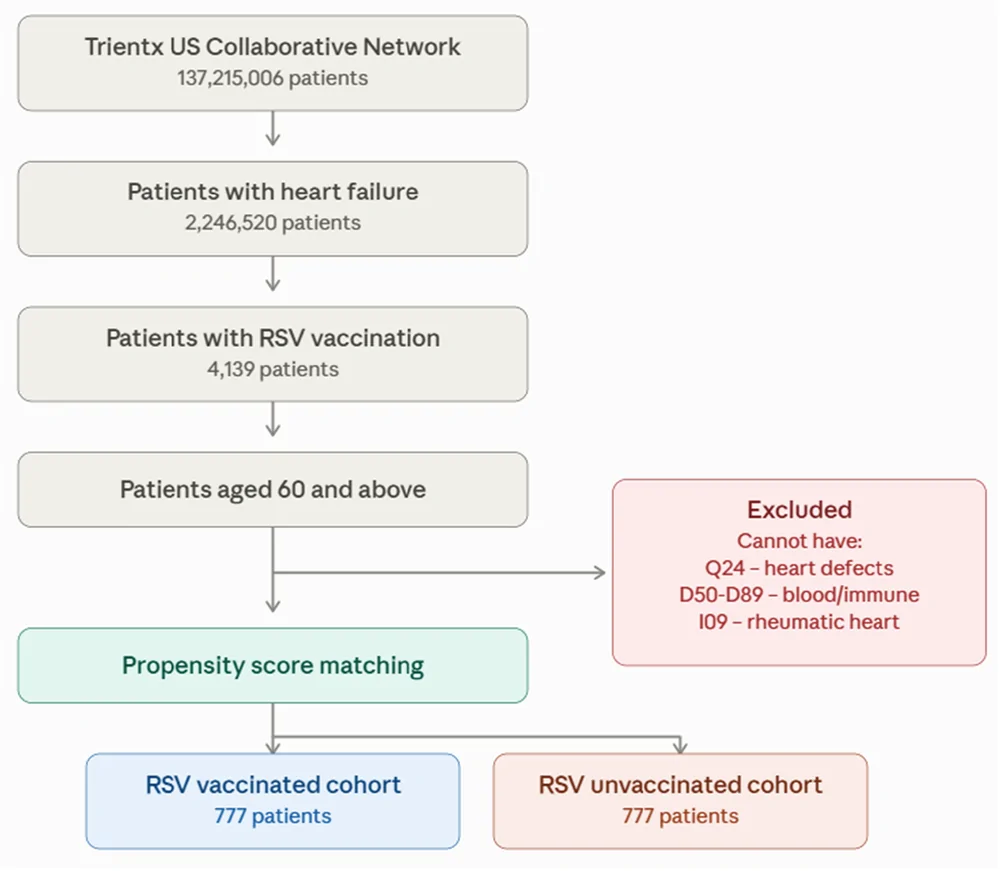
CONSORT flow diagram for the study.

**Table 2:** Baseline population characteristics after propensity score matching.

Category	Variable/medication	Unvaccinated Mean ± SD	Vaccinated Mean ± SD	Unvaccinated patients	Vaccinated patients	*p* value	Std diff
Demographics	Current age	71.3 ± 8.6	71.4 ± 8.6	777	777	0.847	0.012
	Age at index	70.6 ± 8.78	70.6 ± 8.6	777	777	0.933	0.004
	White	—	—	588	601	0.437	0.039
	Female	—	—	359	371	0.541	0.031
	Male	—	—	417	406	0.576	0.028
Diagnosis	Hypertensive diseases (I10‐I1A)	—	—	725	712	0.211	0.063
	Ischemic heart diseases (I20‐I25)	—	—	465	444	0.279	0.055
	Cerebrovascular diseases (I60‐I69)	—	—	174	168	0.713	0.019
	Diabetes mellitus (E08‐E13)	—	—	359	359	1.00	< 0.001
	Chronic lower respiratory diseases (J40‐J4A)	—	—	385	371	0.477	0.036
	Chronic kidney disease (N18)	—	—	188	184	0.812	0.012
	Atrial fibrillation and flutter (I48)	—	—	251	241	0.586	0.028
	Overweight and obesity (E66)	—	—	441	452	0.573	0.029
	Disorders of lipoprotein metabolism (E78)	—	—	686	681	0.697	0.019
	Homelessness (Z59.0)	—	—	10	10	1	< 0.001
	Unemployment, unspecified (Z56.0)	—	—	10	10	1	< 0.001
Medication	Anticoagulants (BL110)	—	—	505	489	0.398	0.075
	Beta blockers/related (CV100)	—	—	617	593	0.143	0.074
	Diuretics (CV700)	—	—	564	565	0.956	0.002
	ACE inhibitors (CV800)	—	—	390	394	0.839	0.010
	Angiotensin II inhibitor (CV805)	—	—	372	359	0.509	0.034
	Oral hypoglycemic agents (HS502)	—	—	330	350	0.307	0.052

**Figure 2:**
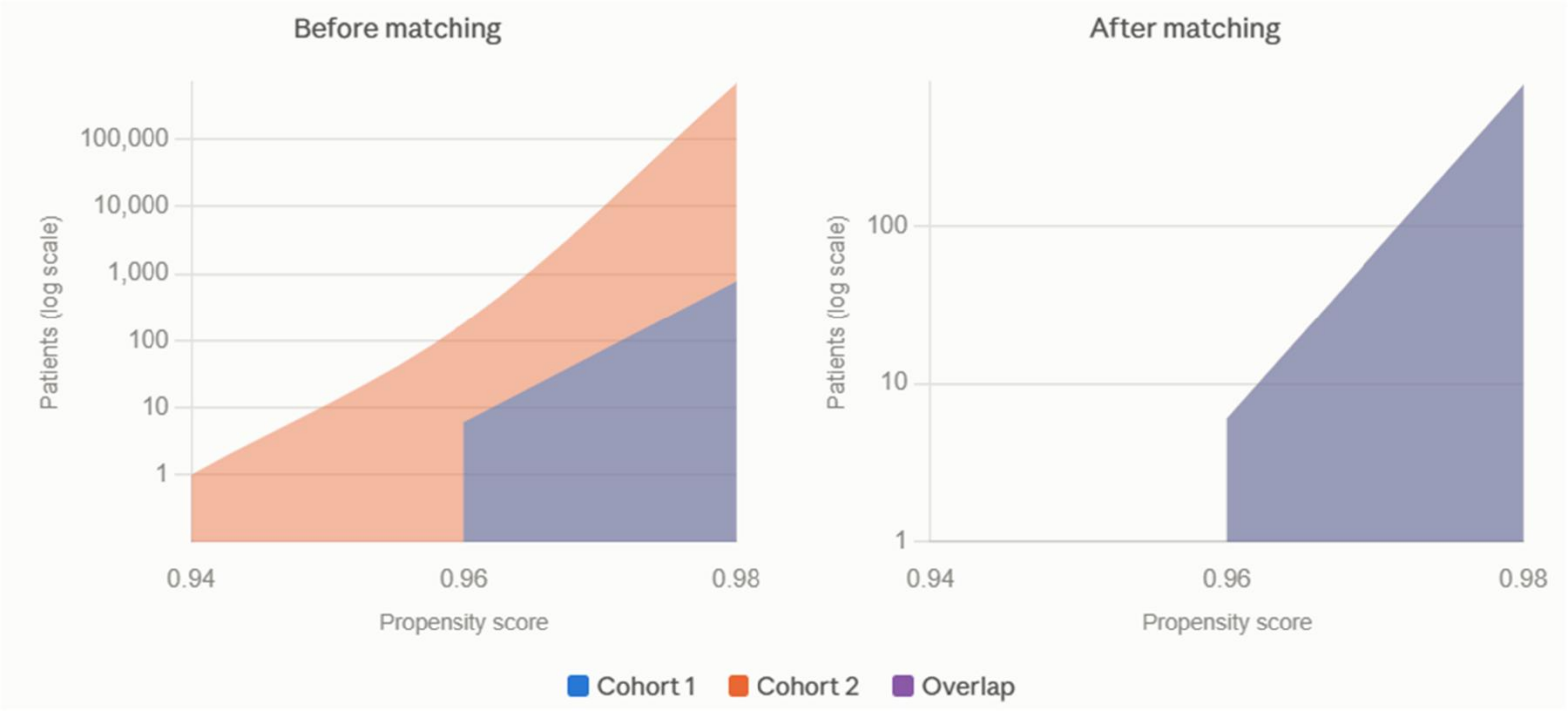
Before and after PSM distribution of both cohorts. Cohort 1 is RSV‐vaccinated, and Cohort 2 is the unvaccinated group.

**Figure 3:**
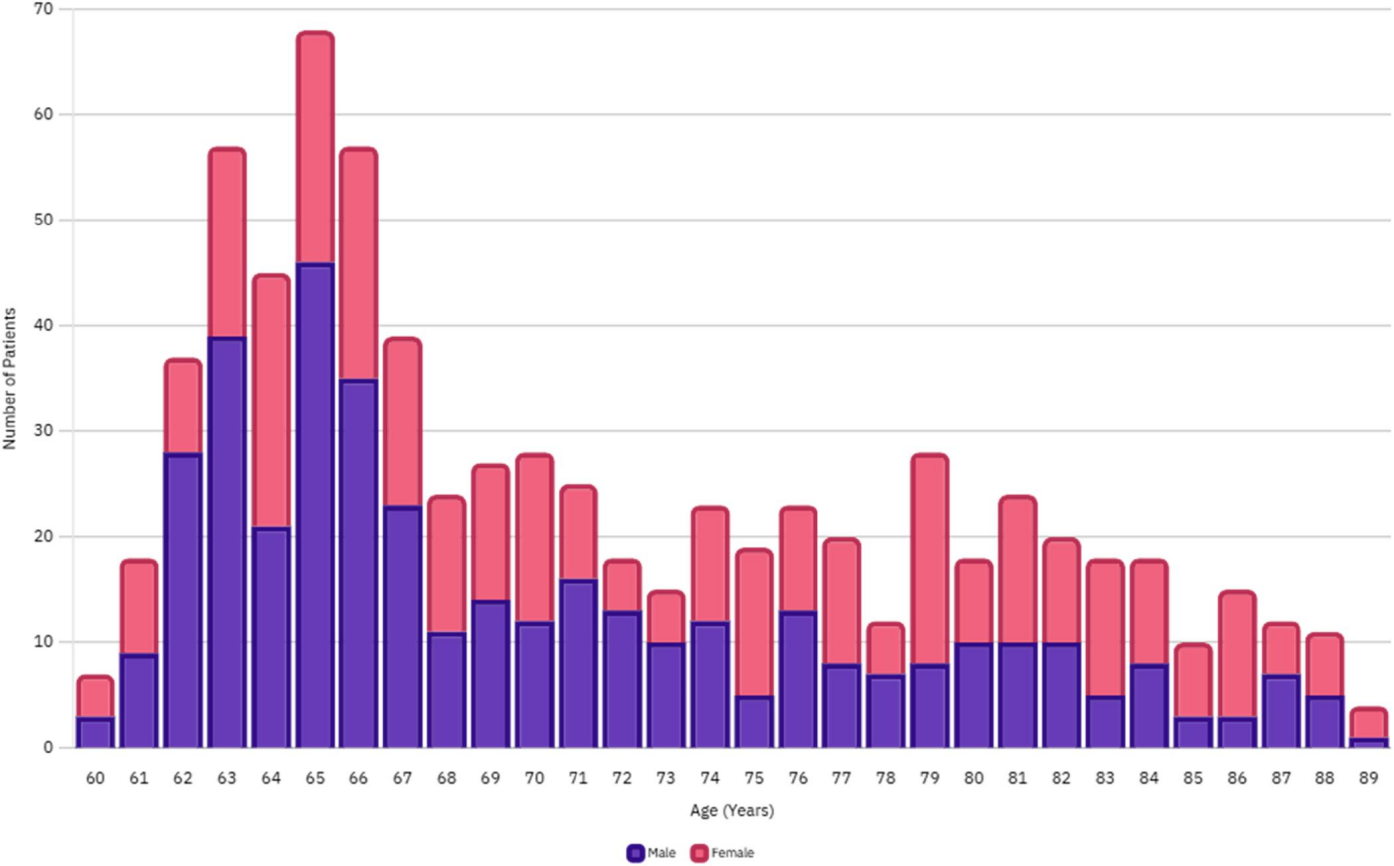
Age distribution of patients included in the study. Patients aged 90+ were 37 in total.

All‐cause mortality (Figure 4) occurred in 3.8% of vaccinated patients and 11.1% of unvaccinated patients, corresponding to a RDs of 7.2% (*p* = 0.001) and an HR of 0.51 (95% CI, 0.33−0.79). Respiratory failure occurred in 7.3% of vaccinated and 9.4% of unvaccinated patients; although numerically lower in the vaccinated group, the difference was not statistically significant (HR 0.87, 95% CI 0.61−1.23).

**Figure 4:**
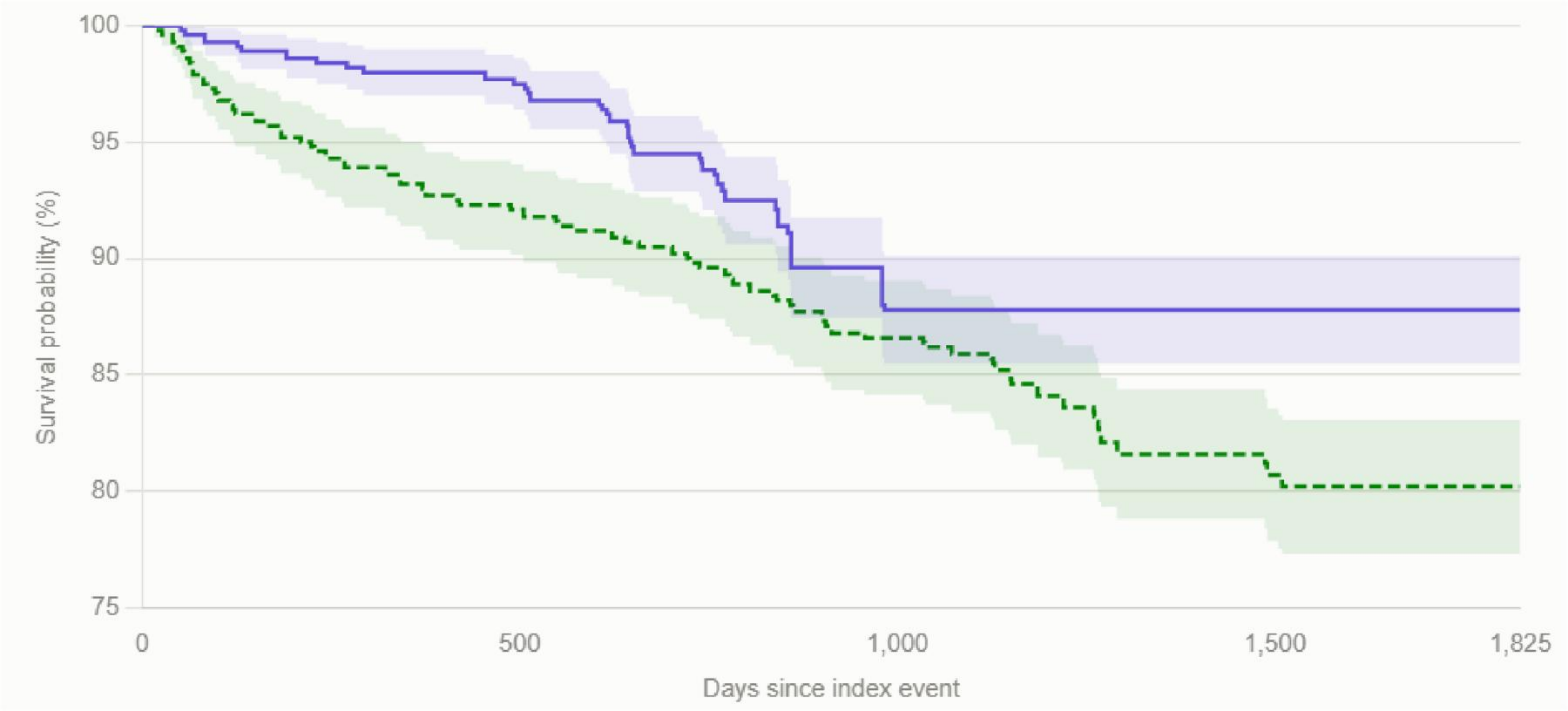
Kaplan−Meier curves for all‐cause mortality by RSV vaccination status in heart failure patients. Follow‐up ran from Day 14 to Day 1825 post‐index; shaded bands show 95% CIs. Survival at end of follow‐up: 87.8% (RSV vaccinated, blue) versus 80.2% (no RSV vaccination, green).

Healthcare encounters favored the vaccinated cohort. Inpatient or emergency visits were numerically lower in vaccinated patients but not statistically significant (29.9% vs. 35.1%, HR 0.97, 95% CI 0.81−1.16). ICU admissions were also reduced (2.7% vs. 5.8%), achieving statistical significance in survival analysis (HR 0.46, 95% CI 0.25−0.86). In contrast, overall healthcare utilization was higher in the vaccinated group (HR 1.85, 95% CI 1.65−2.08, *p* < 0.001), reflecting increased engagement with outpatient or non‐acute services.

No significant differences were observed in major cardiovascular outcomes, including HF exacerbations (HR 1.028, 95% CI 0.89−1.18), myocardial infarction (HR 1.26, 95% CI 0.86−1.84), and stroke (HR 0.96, 95% CI 0.72−1.28). These findings suggest that RSV vaccination may reduce respiratory‐related morbidity and acute care utilization in HF patients without significantly altering cardiovascular event rates.

### 3.1 Subgroup Analysis: Patients Aged 60−75

After PSM, the age 60−75 subgroup included 504 RSV‐vaccinated and 504 unvaccinated HF patients. Mean age at index was 65.1 ± 3.84 years (median 64, IQR 6) in the vaccinated cohort and 65.3 ± 4.53 years (median 65, IQR 7) in the unvaccinated cohort (*p* = 0.3375). Sex distribution was nearly identical between cohorts (58.5% male, 41.5% female in both), and racial composition was similar, with 77.6% of vaccinated and 75.0% of unvaccinated patients identifying as White (*p* = 0.3357).

No significant differences were observed in major cardiovascular outcomes. HF exacerbations occurred in 53.2% of vaccinated and 50.6% of unvaccinated patients (HR 1.127, 95% CI 0.947−1.341). Myocardial infarction occurred in 7.5% versus 7.7% of patients (HR 1.234, 95% CI 0.772−1.972), and stroke occurred in 9.5% versus 10.9% (HR 1.031, 95% CI 0.691−1.538).

Respiratory failure occurred in 6.3% of vaccinated and 10.5% of unvaccinated patients; although numerically lower in the vaccinated group, the difference was not statistically significant (HR 0.730, 95% CI 0.464−1.148).

Inpatient care events were similar between cohorts (30.0% vs. 32.1%; HR 1.103, 95% CI 0.876−1.389). Overall, healthcare utilization was significantly higher in the vaccinated cohort (90.5% vs. 65.9%; HR 2.045, 95% CI 1.764−2.370).

### 3.2 Subgroup Analysis: Patients Aged 75−90

After PSM, the age 75−90 subgroup included 288 RSV‐vaccinated and 288 unvaccinated HF patients. Mean age at index was 80.6 ± 5.13 years (median 80, IQR 7) in the vaccinated cohort and 80.8 ± 5.41 years (median 80, IQR 8) in the unvaccinated cohort (*p* = 0.5586). Sex distribution was nearly identical between cohorts (60.4% female, 39.6% male in both), and racial composition was similar, with 81.9% of vaccinated and 84.0% of unvaccinated patients identifying as White (*p* = 0.5058).

No significant differences were observed in major cardiovascular outcomes. HF exacerbations occurred in 52.1% of vaccinated and 52.4% of unvaccinated patients (HR 0.909, 95% CI 0.724−1.141). Myocardial infarction occurred in 7.6% versus 9.0% of patients (HR 1.058, 95% CI 0.582−1.925), and stroke occurred in 14.6% versus 17.4% (HR 0.999, 95% CI 0.651−1.532).

Respiratory failure occurred in 9.0% of vaccinated and 11.8% of unvaccinated patients; although numerically lower in the vaccinated group, the difference was not statistically significant (HR 0.966, 95% CI 0.560−1.667).

Inpatient care events were numerically lower in the vaccinated cohort (29.5% vs. 40.6%) but did not reach statistical significance (HR 0.865, 95% CI 0.645−1.160). In contrast, overall healthcare utilization was significantly higher in the vaccinated cohort (88.9% vs. 66.3%; HR 1.826, 95% CI 1.504−2.218).

## 4 Discussion

In this propensity‐matched analysis of a large federated electronic health record network, older adults with HF who received an RSV vaccine had substantially lower all‐cause mortality (3.8% vs. 11.1%; RDs 7.2%; *p* = 0.001; HR 0.51, 95% CI 0.33–0.79) and fewer intensive care unit admissions (2.7% vs. 5.8%; HR 0.46, 95% CI 0.25–0.86) than matched unvaccinated patients (*n* = 777 per arm). Inpatient or emergency encounters were numerically lower but were no longer statistically significant (29.9% vs. 35.1%; HR 0.97, 95% CI 0.81–1.16). Respiratory failure was numerically less frequent but did not reach statistical significance (7.3% vs. 9.4%; HR 0.87, 95% CI 0.61–1.23), and we found no differences in HF exacerbation, myocardial infarction, or stroke (HF exacerbation HR 1.028, 95% CI 0.89–1.18; myocardial infarction HR 1.26, 95% CI 0.86–1.84; stroke HR 0.96, 95% CI 0.72–1.28). Paradoxically, overall healthcare utilization was *higher* in the vaccinated cohort (HR 1.85, 95% CI 1.65–2.08; *p* < 0.001). Age‐stratified analyses in patients aged 60–75 and 75–90 years were directionally consistent with the overall matched cohort. Taken together, these updated results are best read as a hypothesis‐generating signal that RSV vaccination is associated with lower all‐cause mortality and less critical‐care use in a high‐risk cardiac population, without a confirmed reduction in all‐cause inpatient or emergency encounters, and, just as importantly, as a reminder of how strongly health‐seeking behavior shapes what we observe in real‐world vaccine data.

### 4.1 All‐Cause Mortality and Kaplan–Meier Survival

All‐cause mortality was the prespecified primary outcome and is statistically significant in the updated matched cohort, favoring RSV vaccination. Death occurred in 3.8% of vaccinated patients and 11.1% of unvaccinated patients, a 7.2 percentage‐point absolute RD (*p* = 0.001) and an approximately 49% lower hazard of death (HR 0.51, 95% CI 0.33–0.79).

Kaplan–Meier curves for all‐cause mortality (Figure 4) were constructed from Day 14 to Day 1825 after the index date, with shaded 95% confidence bands. The curves separated in favor of vaccination and remained apart through the end of follow‐up: estimated survival was 87.8% among RSV‐vaccinated patients compared with 80.2% among unvaccinated patients. Commencing follow‐up at Day 14 reduces the contribution of very early deaths that cannot plausibly be attributed to vaccine effect and lessens a component of immortal‐time bias around the vaccination encounter. Concordance of the crude RDs, the Cox proportional hazards model, and the Kaplan–Meier analysis indicates that the mortality association is not an artifact of a single estimator.

The magnitude of the mortality signal, however, deserves scrutiny. The pivotal randomized trials of RSVPreF3 OA and RSVpreF demonstrated efficacy of 67%−83% against RSV‐associated lower respiratory tract disease in adults aged ≥ 60 years, including those with cardiorespiratory comorbidity. Still, neither trial was powered for, nor demonstrated, a reduction in all‐cause mortality [6, 7]. Real‐world target trial emulation in US veterans similarly showed high effectiveness against RSV‐specific outcomes (78% against documented infection, 80% against RSV‐associated hospitalization) without evidence of a broad, nonspecific survival benefit [10]. Because RSV accounts for only a fraction of deaths in an HF population, an approximately 49% relative and 7.2 percentage‐point absolute reduction in all‐cause mortality over the observation window remains larger than the attributable RSV burden can readily explain on its own. Residual confounding, including the healthy‐vaccinee effect, may therefore contribute to part of the observed survival difference. That caveat notwithstanding, the updated analysis demonstrates a statistically significant mortality reduction that favors RSV vaccination and is internally consistent across risk, hazard, and Kaplan–Meier estimators. The finding should be interpreted as a robust observational association, not as proof of a causal, vaccine‐attributable survival benefit.

### 4.2 Biological and Clinical Plausibility

There are good reasons to expect that preventing RSV matters to patients with HF. RSV is now recognized as a major cause of medically attended acute respiratory illness in older adults, with an estimated 470 000 hospitalizations and 33 000 in‐hospital deaths annually among adults aged ≥ 60 years in high‐income countries [11], and it produces illness of comparable or greater severity to influenza in elderly and high‐risk patients [1]. Cardiopulmonary comorbidity is present in 45%−63% of adults hospitalized with RSV, and 14%−22% of such admissions are complicated by a cardiovascular event [5]. In the largest surveillance study to date, 22% of hospitalized adults aged ≥ 50 years with laboratory‐confirmed RSV experienced an acute cardiac event, most commonly acute HF (16%), and those events nearly doubled the risk of ICU admission, mechanical ventilation, or in‐hospital death [12]. A vaccine that averts even a minority of RSV episodes in patients with limited cardiopulmonary reserve could therefore plausibly translate into fewer critical‐care admissions and fewer deaths, which is the pattern now observed, even though a broader reduction in inpatient or emergency presentations was not confirmed.

### 4.3 Age‐Stratified Subgroup Analyses

The updated analysis included age‐stratified, 1:1 propensity‐matched subgroups to examine whether the overall pattern varied by age.

Among patients aged 60–75 years (504 vaccinated and 504 unvaccinated), no significant differences were observed in major cardiovascular outcomes. HF exacerbations occurred in 53.2% of vaccinated and 50.6% of unvaccinated patients (HR 1.127, 95% CI 0.947–1.341). Myocardial infarction occurred in 7.5% versus 7.7% (HR 1.234, 95% CI 0.772–1.972), and stroke occurred in 9.5% versus 10.9% (HR 1.031, 95% CI 0.691–1.538). Respiratory failure was numerically lower in the vaccinated group (6.3% vs. 10.5%) but was not statistically significant (HR 0.730, 95% CI 0.464–1.148). Inpatient care events were similar (30.0% vs. 32.1%; HR 1.103, 95% CI 0.876–1.389). Overall healthcare utilization was significantly higher after vaccination (90.5% vs. 65.9%; HR 2.045, 95% CI 1.764–2.370).

Among patients aged 75–90 years (288 vaccinated and 288 unvaccinated), cardiovascular outcomes were likewise null. HF exacerbations occurred in 52.1% versus 52.4% (HR 0.909, 95% CI 0.724–1.141), myocardial infarction in 7.6% versus 9.0% (HR 1.058, 95% CI 0.582–1.925), and stroke in 14.6% versus 17.4% (HR 0.999, 95% CI 0.651–1.532). Respiratory failure did not differ significantly (9.0% vs. 11.8%; HR 0.966, 95% CI 0.560–1.667). Inpatient encounters were numerically lower in vaccinated patients (29.5% vs. 40.6%) but did not reach statistical significance (HR 0.865, 95% CI 0.645–1.160) and should not be described as a confirmed benefit in this age band. Overall healthcare utilization was again significantly higher (88.9% vs. 66.3%; HR 1.826, 95% CI 1.504–2.218).

These subgroups indicate that neutral cardiovascular event rates, nonsignificant differences in respiratory failure, and higher overall healthcare utilization are consistent across age bands rather than being driven by a single stratum. All‐cause mortality and ICU admission were not reported separately within age strata, so effect modification by age for the primary survival endpoint cannot be inferred from the present data.

### 4.4 Interpreting the Divergent Utilization Signals

The previous finding of significantly fewer inpatient or emergency encounters is not confirmed in the updated analysis (29.9% vs. 35.1%; HR 0.97, 95% CI 0.81–1.16) and should no longer be presented as a benefit of vaccination. What remains is a dissociation between significantly lower all‐cause mortality and ICU admission on the one hand, and significantly greater overall healthcare utilization on the other. That pattern is internally informative. Patients who present for a discretionary preventive vaccine are, almost by definition, engaged with the health system: they attend clinic, have documented medication lists, and are more likely to have their chronic disease titrated and monitored. Higher outpatient and non‐acute contact is a marker of that engagement, and greater engagement independently predicts fewer unplanned admissions in HF, where guideline‐directed medical therapy, volume management, and early decompensation review are the principal determinants of hospitalization [13]. In other words, the vaccinated cohort may be advantaged not only by the vaccine but by the care pathway that delivered it. Because a federated EHR network captures encounter‐level codes rather than functional status, matching on diagnoses and prescriptions alone cannot remove this behavioral gradient. The strengthened utilization association (HR 1.85, 95% CI 1.65–2.08) was reproduced in both age subgroups and is therefore unlikely to be a chance finding confined to the overall cohort. A reduction in ICU admission (HR 0.46, 95% CI 0.25–0.86) without a parallel reduction in all‐cause inpatient or emergency visits may indicate that vaccination, or the care associated with it, is more closely linked to prevention of the most severe decompensations than to prevention of all unplanned encounters.

### 4.5 Relation to the Wider Literature on Respiratory Vaccination in Cardiac Disease

Our findings sit within an older and unresolved debate about whether respiratory vaccination modifies cardiovascular outcomes. The IAMI trial showed that influenza vaccination given early after myocardial infarction reduced the composite of death, MI, or stent thrombosis and halved cardiovascular mortality at 12 months [14]. In contrast, IVVE, a multinational randomized trial in patients with symptomatic HF, found no reduction in its primary composite cardiovascular endpoint with influenza vaccination, although pneumonia and hospitalization were reduced during periods of viral circulation [15]. That dissociation, infection‐related benefit without a clear cardiovascular‐event benefit, partially mirrors what we now report here for RSV: significant reductions in all‐cause mortality and critical‐care use, with neutral effects on HF exacerbation, MI, and stroke, and without a significant reduction in all‐cause inpatient or emergency encounters. This coherence across pathogens and data sets strengthens the argument that the benefit of respiratory vaccination in HF is mediated mainly through averted infection and averted decompensation‐triggering illness, rather than through direct modification of atherothrombotic risk.

### 4.6 Limitations

Several limitations temper our conclusions. First, and most importantly, this is a non‐randomized analysis and vulnerable to the healthy vaccinee effect, a bias repeatedly demonstrated in observational influenza vaccine studies, in which vaccinated individuals show apparent survival advantages that persist outside periods of viral circulation and are therefore not attributable to vaccination [16]. Propensity score matching achieved excellent balance across the measured demographic, comorbidity, and medication covariates in Table 2 (all standardized differences < 0.1), but balance on measured covariates provides no guarantee of balance on unmeasured ones [17]. Left ventricular ejection fraction, NYHA functional class, natriuretic peptide concentrations, frailty, cognitive status, nutritional state, socioeconomic position, do‐not‐resuscitate or hospice status, and completeness of guideline‐directed therapy were not available for matching, and each plausibly favors the vaccinated group.

Second, our outcomes were nonspecific. We could not confirm RSV infection by laboratory testing, so we cannot demonstrate that the events prevented were RSV events; the analysis is agnostic as to mechanism. Third, exposure was defined by any documented RSV vaccination code from May 1, 2023, to January 1, 2026, without alignment to RSV seasonality and without a uniform time zero relative to disease severity; such designs are susceptible to immortal time and index‐date bias, which systematically favor the treated group [18]. Follow‐up for the mortality Kaplan–Meier analysis began at Day 14, which reduces but does not eliminate these biases. A negative‐control outcome analysis and a seasonal (in‐season vs. out‐of‐season) comparison would help quantify how much of the mortality signal is genuine, and we regard these as essential next steps. Fourth, 4139 HF patients met initial eligibility criteria, and 777 vaccinated and 777 unvaccinated patients were retained after matching. Documented RSV vaccination among older adults with HF remains limited, reflecting both genuinely low national uptake, approximately 38%−42% coverage even after the 2024 ACIP recommendation for all adults ≥ 75 years and at‐risk adults 60−74 years [19, 20], and incomplete capture of pharmacy‐administered vaccinations in health‐system EHRs. Vaccinated patients whose immunization was recorded only in a retail pharmacy or state registry would have been misclassified as unvaccinated, a differential misclassification that also plausibly enriches the “unvaccinated” arm with less engaged patients.

Fifth, absolute event rates for death (3.8% vs. 11.1%) and ICU admission (2.7% vs. 5.8%) remain modest, so estimates are imprecise, CIs are relatively wide, and an approximate number needed to vaccinate of 14, derived from the 7.2% absolute RDs for mortality, should be regarded as an unstable descriptive figure rather than a usable clinical statistic. Sixth, multiple outcomes were tested without formal correction for multiplicity, so some findings may represent chance. Additionally, the use of I50.x diagnostic codes within the preceding 5 years may have captured a heterogeneous HF population, including patients with remote or clinically stable disease, and the analysis did not distinguish among HF phenotypes such as HFrEF, HFmrEF, and HFpEF. Other potentially important markers of healthcare engagement and preventive‐care behavior, including influenza, pneumococcal, and COVID‐19 vaccination, as well as the frequency of primary‐care and cardiology follow‐up, were not incorporated into the analysis and may represent additional sources of residual confounding. Furthermore, vaccination timing was not analyzed in sufficient detail by calendar month or RSV season, limiting assessment of the relationship between vaccination timing, seasonal RSV circulation, and subsequent outcomes. Although an age‐stratified analysis was performed among patients aged 75–90 years, measures of frailty and functional status were not available to determine whether the findings were consistent across different levels of biological vulnerability within this older population. Finally, because cause‐specific mortality was not reliably available, we could not determine whether observed deaths were cardiovascular, respiratory, RSV‐related, or attributable to other causes.

Finally, TriNetX aggregates de‐identified, harmonized EHR data from participating US healthcare organizations [21]; while this affords scale and diagnostic granularity, it cannot capture care delivered outside the network, cause‐specific mortality, or out‐of‐hospital deaths, and coding practices vary between contributing sites.

## 5 Conclusions

Our analysis provides real‐world evidence that RSV vaccination in older adults with HF is associated with significantly lower all‐cause mortality and fewer critical‐care encounters, with no signal of cardiovascular harm, a reassuring finding for a population in which vaccination decisions are often deferred because of clinical fragility. Inpatient or emergency encounters and respiratory failure were not significantly reduced. Given the demonstrated frequency of acute cardiac events during RSV hospitalization [12] and the persistently low vaccination coverage among exactly the patients most likely to benefit [19, 20], HF clinics represent an underused and logical setting for opportunistic RSV immunization. The mortality association is statistically significant and favors vaccination, but it is observational and may be inflated by residual confounding; it should not yet be treated as a causal estimate for counseling or cost‐effectiveness modeling. Confirmation will require analyses that use RSV‐specific endpoints, season‐restricted comparisons, negative‐control outcomes, active‐comparator designs (e.g., influenza‐vaccinated patients as the reference group), and ideally, prospective evaluation in patients with well‐characterized HF phenotypes.

## Author Contributions

A.K. was involved with the conceptualization of the study, original write‐up, methodology, review, and editing of the manuscript, and visualization of the study. M.R.S. was involved with the conceptualization of the study, reviewing and editing the manuscript, data analysis, and investigation of data. J.N. was involved with the original write‐up, reviewing, and editing of the manuscript. M.I. was involved with the original write‐up of the study. H.T., M.F., R.J.M., and G.C.F. were involved with supervision, validation, methodology, and review and editing of the manuscript.

## Funding

The authors have nothing to report.

## Ethics Statement

This study was conducted using the TriNetX Research Network, a federated, de‐identified electronic health record database. All data were de‐identified in accordance with the Health Insurance Portability and Accountability Act (HIPAA), and no protected health information was accessed. As a result, this study was determined to be exempt from institutional review board approval and informed consent requirements.

## Conflicts of Interest

Dr Marat Fudim was supported by the NIH, Alleviant, Gradient, Novo Nordisk, Reprieve, Sardocor, Tenax, and Doris Duke. He is a consultant and/or has ownership interest in Abbott, Acorai, Ajax, Alio Health, Alleviant, Artha, Astellas, Audicor, AxonTherapies, Berlin Heals, Bioventrix, BMS, Bodyguide, Boehringer Ingelheim, Bodyport, Boston Scientific, Broadview, Cadence, Cardiosense, Cardioflow, Corstasis, Clinical Accelerator, CVRx, Daxor, Edwards LifeSciences, Echosens, Feldschuh Foundation, Fire1, FlowMod, FutureCardia, Gradient, Hatteras, HemodynamiQ, Impulse Dynamics, ISHI, Lumia Health, Medtronic, Novo Nordisk, NucleusRx, Omega, Orchestra, Paragate, Parasym, Pharmacosmos, Privebio, Procyreon, Proton Intelligence, Puzzle, ReCor, SalubrisBio, Scirent, SCPharma, Shifamed, Splendo, Summacor, SutroSam, SyMap, Terumo, Tricav, Vifor Pharma, Vasa, Vironix, Viscardia, VizAI, Zoll. Dr. Robert J. Mentz received research support and/or honoraria from Abbott, Alleviant Medical, American Regent, Amgen, AstraZeneca, Bayer, Boehringer Ingelheim, CVRx, Cytokinetics, Daiichi Sankyo, Edwards, Eli Lilly, Endotronix, Lexicon, Medtronic, Merck, Novartis, Novo Nordisk, Otsuka, Pfizer, Pharmacosmos, Reprieve Cardiovascular, Respicardia, Roche, Rocket Pharmaceuticals, Vifor, Windtree Therapeutics, and Zoll. Dr Gregg C. Fonarow reports consulting for Abbott, Amgen, AstraZeneca, Bayer, Boehringer Ingelheim, Cytokinetics, Eli Lilly, Johnson & Johnson, Medtronic, Merck, Novartis, and Pfizer. The other authors declare no conflicts of interest.

## Data Availability

Due to data use agreements and patient privacy considerations, the raw data cannot be publicly shared. However, researchers with institutional affiliations can request access to the TriNetX platform at www.trinetx.com.

## References

[1] A. R. Falsey , P. A. Hennessey , M. A. Formica , C. Cox , and E. E. Walsh , “Respiratory Syncytial Virus Infection in Elderly and High‐Risk Adults,” New England Journal of Medicine 352, no. 17 (2005): 1749–1759, 10.1056/NEJMoa043951. 15858184

[2] T. Shi , A. Denouel , A. K. Tietjen , et al., “Global Disease Burden Estimates of Respiratory Syncytial Virus–Associated Acute Respiratory Infection in Older Adults in 2015: A Systematic Review and Meta‐Analysis,” supplement, Journal of Infectious Diseases 222, no. S7 (2020): S577–S583, 10.1093/infdis/jiz059. 30880339

[3] B. Ackerson , H. F. Tseng , L. S. Sy , et al., “Severe Morbidity and Mortality Associated With Respiratory Syncytial Virus Versus Influenza Infection in Hospitalized Older Adults,” Clinical Infectious Diseases 69, no. 2 (2019): 197–203, 10.1093/cid/ciy991. PMC6603263 30452608

[4] Y. Penders , A. R. Falsey , D. L. Bhatt , et al., “Burden and Severity of Respiratory Syncytial Virus Infection in Adults With Cardiovascular Diseases: A Systematic Literature Review,” Progress in Cardiovascular Diseases 92 (2025): 130–143, 10.1016/j.pcad.2025.06.010. 40614914

[5] K. S. Ivey , K. M. Edwards , and H. K. Talbot , “Respiratory Syncytial Virus and Associations With Cardiovascular Disease in Adults,” Journal of the American College of Cardiology 71, no. 14 (2018): 1574–1583, 10.1016/j.jacc.2018.02.013. 29622165

[6] A. Papi , M. G. Ison , J. M. Langley , et al., “Respiratory Syncytial Virus Prefusion F Protein Vaccine in Older Adults,” New England Journal of Medicine 388, no. 7 (2023): 595–608, 10.1056/NEJMoa2209604. 36791160

[7] E. E. Walsh , G. Pérez Marc , A. M. Zareba , et al., “Efficacy and Safety of a Bivalent RSV Prefusion F Vaccine in Older Adults,” New England Journal of Medicine 388, no. 16 (2023): 1465–1477, 10.1056/NEJMoa2213836. 37018468

[8] M. Melgar , A. Britton , L. E. Roper , et al., “Use of Respiratory Syncytial Virus Vaccines in Older Adults: Recommendations of the Advisory Committee on Immunization Practices—United States, 2023,” MMWR. Morbidity and Mortality Weekly Report 72 (2023): 793–801, 10.15585/mmwr.mm7229a4. PMC10360650 37471262

[9] “TriNetX Analytics Network Database,” TriNetX, LLC, accessed December 28, 2025, https://trinetx.com/.

[10] K. L. Bajema , L. Yan , Y. Li , et al., “Respiratory Syncytial Virus Vaccine Effectiveness Among US Veterans, September, 2023 to March, 2024: A Target Trial Emulation Study,” Lancet Infectious Diseases 25, no. 6 (2025): 625–633, 10.1016/S1473-3099(24)00796-5. 39848264

[11] M. Savic , Y. Penders , T. Shi , A. Branche , and J. Y. Pirçon , “Respiratory Syncytial Virus Disease Burden in Adults Aged 60 Years and Older in High‐Income Countries: A Systematic Literature Review and Meta‐Analysis,” Influenza and Other Respiratory Viruses 17, no. 1 (2023): e13031, 10.1111/irv.13031. PMC9835463 36369772

[12] R. C. Woodruff , M. Melgar , H. Pham , et al., “Acute Cardiac Events in Hospitalized Older Adults With Respiratory Syncytial Virus Infection,” JAMA Internal Medicine 184, no. 6 (2024): 602–611, 10.1001/jamainternmed.2024.0212. PMC11019447 38619857

[13] P. A. Heidenreich , B. Bozkurt , D. Aguilar , et al., “2022 AHA/ACC/HFSA Guideline for the Management of Heart Failure: A Report of the American College of Cardiology/American Heart Association Joint Committee on Clinical Practice Guidelines,” Circulation 145, no. 18 (2022): 895, 10.1161/CIR.0000000000001063. 35363499

[14] O. Fröbert , M. Götberg , D. Erlinge , et al., “Influenza Vaccination After Myocardial Infarction: A Randomized, Double‐Blind, Placebo‐Controlled, Multicenter Trial,” Circulation 144, no. 18 (2021): 1476–1484, 10.1161/CIRCULATIONAHA.121.057042. 34459211

[15] M. Loeb , A. Roy , H. Dokainish , et al., “Influenza Vaccine to Reduce Adverse Vascular Events in Patients With Heart Failure: A Multinational Randomised, Double‐Blind, Placebo‐Controlled Trial,” Lancet Global Health 10, no. 12 (2022): e1835–e1844, 10.1016/S2214-109X(22)00432-6. 36400089

[16] C. Remschmidt , O. Wichmann , and T. Harder , “Frequency and Impact of Confounding by Indication and Healthy Vaccinee Bias in Observational Studies Assessing Influenza Vaccine Effectiveness: A Systematic Review,” BMC Infectious Diseases 15 (2015): 429, 10.1186/s12879-015-1154-y. PMC4609091 26474974

[17] P. C. Austin , “Balance Diagnostics for Comparing the Distribution of Baseline Covariates Between Treatment Groups in Propensity‐Score Matched Samples,” Statistics in Medicine 28, no. 25 (2009): 3083–3107, 10.1002/sim.3697. PMC3472075 19757444

[18] S. Suissa , “Immortal Time Bias in Pharmacoepidemiology,” American Journal of Epidemiology 167, no. 4 (2008): 492–499, 10.1093/aje/kwm324. 18056625

[19] A. Britton , L. E. Roper , C. N. Kotton , et al., “Use of Respiratory Syncytial Virus Vaccines in Adults Aged ≥60 Years: Updated Recommendations of the Advisory Committee on Immunization Practices—United States, 2024,” MMWR. Morbidity and Mortality Weekly Report 73, no. 32 (2024): 696–702, 10.15585/mmwr.mm7332e1. 39146277

[20] E. M. La , C. B. McGuiness , D. Singer , M. Yasuda , and C. C. Chen , “RSV Vaccination Uptake Among Adults Aged 60 Years and Older in the United States During the 2023–2025 Vaccination Seasons,” Human Vaccines & Immunotherapeutics 21, no. 1 (2025): 2535755, 10.1080/21645515.2025.2535755. PMC12326572 40763207

[21] M. B. Palchuk , J. W. London , D. Perez‐Rey , et al., “A Global Federated Real‐World Data and Analytics Platform for Research,” JAMIA Open 6, no. 2 (2023): ooad035, 10.1093/jamiaopen/ooad035. PMC10182857 37193038

